# Role of imaging in the management of thyroglossal duct cyst carcinomas (TGC-TIRADS): a single centre retrospective study over 16 years

**DOI:** 10.3389/fonc.2023.1201774

**Published:** 2023-11-23

**Authors:** Abhishek Mahajan, Shonal Deokar, Shubham Suryavanshi, Ujjwal Agarwal, Richa Vaish, Shreya Shukla, Tanvi Vaidya, Vasundhara Smriti, Vidisha Tuljapurkar, Shivam Rastogi, Atif Shaikh, Pankaj Chaturvedi, Anil Keith Dcruz, Asawari Patil, Munita Bal, Swapnil Ulhas Rane, Neha Mittal, Vanita Noronha, Kumar Prabhash, Vijay Patil, Prathamesh Pai

**Affiliations:** ^1^ Department of Radiology, The Clatterbridge Cancer Centre, Liverpool, United Kingdom; ^2^ Department of Radiology, Head and Neck Surgical Oncology, Medical Oncology, Pathology, Tata Memorial Hospital, Mumbai, India

**Keywords:** Thyroglossal cyst carcinoma, Thyroglossal duct cyst, papillary carcinoma, TGC-TIRADS, thyroglossal cyst imaging, thyroglossal cyst management

## Abstract

**Introduction:**

Thyroglossal duct cyst (TGDC) is the most frequently encountered developmental anomaly in thyroid genesis with a reported incidence of 7% in the adult population. The cyst is known to develop anywhere along the pathway of thyroid descent but is more frequently seen in the infrahyoid neck in the midline. The incidence of malignancy in a TGDC is approximately 1%; a majority of these are papillary carcinomas. This study was conducted at a single tertiary care centre which spanned over a decade which adds practice changing evidence-based knowledge to existing literature on this rare entity. A comprehensive study which conclusively establishes the imaging features predictive of malignancy in TGDC carcinomas (TGDCa), the protocol for optimal management, clinical outcome and long-term survival of these patients is not available. Although TGDC carcinoma is thought to have an excellent prognosis, there is not enough data available on the long-term survival of these patients. The aim of this study was to identify whether neck ultrasound (US) can serve as an accurate imaging tool for the preoperative diagnosis of TGDC carcinomas.

**Methods:**

We accessed the electronic medical records of 86 patients with TGDC between January 2005 to December 2021. Of these, 22 patients were detected with TGDC papillary carcinoma on histopathologic examination. Relevant imaging, treatment and follow up information for all cases of TGDC carcinoma were retrospectively reviewed. We compared US characteristics predictive of malignancy across outcomes groups; malignant vs benign using the Chi-square test. Based on the results, a TGC-TIRADS classification was proposed with calculation of the percentage likelihood of malignancy for each category.

**Results:**

Compared to benign TGDCs, malignant TGDCs were more likely to present with following US characteristics: irregular or lobulated margins (90.40 vs. 38.10%), solid-cystic composition (61.90 vs. 17.07%), internal vascularity (47.62 vs. 4.88 %), internal calcification (76.19 vs. 7.32 %) (each p value < 0.005). Calcifications and internal vascularity were the most specific while irregular/lobulated margins were the most sensitive feature for malignancy. AUC under the ROC curve was 0.88. Allpatients were operated and were disease free at the end of 5 years or till the recent follow up.

**Discussion:**

US is the imaging modality of choice for pre-operative diagnosis of TGDC carcinoma. Thepre-operative diagnosis and risk stratification of thyroglossal lesions will be aided by the application of the proposed TGC-TIRADS classification, for which the percentage likelihood of malignancy correlated well with the results in our study. Sistrunk procedure is adequate for isolated TGDC carcinoma; suspicious neck nodes on imaging also necessitates selective nodal dissection. Papillary carcinomas have an excellent prognosis with low incidence of disease recurrence.

## Introduction

Thyroglossal duct cyst (TGDC) is the most frequently encountered developmental anomaly in thyroid genesis with a reported incidence of 7% in the adult population (1). The cyst is known to develop anywhere along the pathway of thyroid descent but is more frequently seen in the infra-hyoid neck in the midline. The incidence of malignancy in a TGDC is approximately 1%; a majority of these are papillary carcinomas (1, 2).

This is a rare entity and this study was conducted at a single tertiary care centre which spanned over a decade which adds practice changing evidence based knowledge to existing literature on this rare entity. This is one of the largest series where imaging features have been studied. A comprehensive study which conclusively establishes the imaging features predictive of malignancy in TGDC carcinomas (TGDCa), the protocol for optimal management, clinical outcome and long-term survival of these patients is not available. Consequently, providing patients and clinicians with pertinent information with respect to the role of imaging, further investigations, management and prognosis poses a challenge. Although TGDC carcinoma is thought to have an excellent prognosis (3), there is not enough data available on the long-term survival of these patients.

The aim of this study was to identify whether neck ultrasound (US) can serve as an accurate imaging tool for the pre-operative diagnosis of TGDC carcinomas. We also analyzed the management protocols utilized and their impact on patient outcomes. To the best of our knowledge, this is the largest study of its kind and the first comprehensive study in patients with TGDC carcinomas, which provides evidence-based information regarding the role of imaging, optimal management protocol and clinical outcomes of these cases.

## Materials and methods

The study was approved by the Institutional Review Board (IRB). Waiver consent was obtained for the study. We retrospectively reviewed the electronic medical records (EMR) of 86 patients with TGDC who were referred to our institute between January 2005 to December 2021. Of these, 22 patients were detected with TGDC papillary carcinoma on histopathologic examination. Relevant clinical data, histopathologic details, treatment and follow-up information for all cases of TGDC carcinoma, were accessed from the electronic medical records. US images of all patients were reviewed. US and CT evaluations were studied by two senior radiologists with 10-12 years of experience in head-neck onco imaging. Since all studies were performed by skilled radiologists, the errors were significantly reduced. Inter-observer bias, however, remains as in any other study in which observations are done by more than one observer. We have used different ultrasound machines including GE LOGIQ E7, GE LOGIQ E9 and Samsung RS80 EVO, Philips CV350. Computed tomography (CT) images were reviewed as per their availability. Siemens Somatom Sensation 16 slice CT scanner machine was used with iohexol (Omnipaque) contrast agent. US characteristics which were assessed included: location, composition, margins, internal vascularity, presence of calcifications, internal echoes and lymphadenopathy. On US, microcalcifications (diameter less than 1mm) appear as hyperechoic foci and do not have acoustic shadow. Macrocalcifications (diameter larger than 1mm) show hyperechoic area with posterior acoustic shadowing. Peripheral calcifications are seen when large flecks of calcium deposit in the periphery of the wall. On US, central vascularity within the solid component of the lesion was considered positive for vascularity. Lesions with peripheral vascularity were not included in the positives as benign colloid nodules can show peripheral vascularity. Inspissated colloid (which can mimic solid component) within a cystic nodule will show no central vascularity. Imaging features assessed on CT were: lesion location, density, enhancement characteristics, margins and associated lymphadenopathy.

For surgical patients, histopathological diagnosis was considered the gold standard while for the rest, cytopathology results were considered as the gold standard. Solid component of the lesion was targeted under USG guidance. The adequacy of sample was ensured by the onsite cytologist.

### Statistical analysis

All statistical analyses were performed using SPSS software, version 25. A 2x2 contingency table which included the sonographic characteristics predictive of malignancy was computed. Comparison of categorical factors (across outcomes groups, i.e., malignant vs benign) was done using Chi-square (Fishers Exact Test). A p-value of less than 0.05 was considered statistically significant. Sensitivity, specificity, positive predictive value (PPV), and negative predictive value (NPV) for each imaging feature suspicious of malignancy were calculated. We plotted a receiver operating characteristic (ROC) curve to evaluate the diagnostic accuracy of the US predictors of malignancy.

## Results

### Patient demographics

The median age of presentation of TGDC carcinoma was 47 years with a range of 22 to 48 years. There were 13 males and 9 females, with a 1.4:1 male-to-female gender ratio.

### Clinical presentation

Of the 22 patients with proven TGDC carcinoma, 2 were asymptomatic while the rest 20 patients presented with a palpable, painless neck swelling. The duration of symptoms was 18 to 48 months, with a mean of 24.5 months. On clinical examination, all lesions were mobile on deglutition. No fixity to the overlying skin was noted.

### Ultrasound imaging features

**Figure 1:**
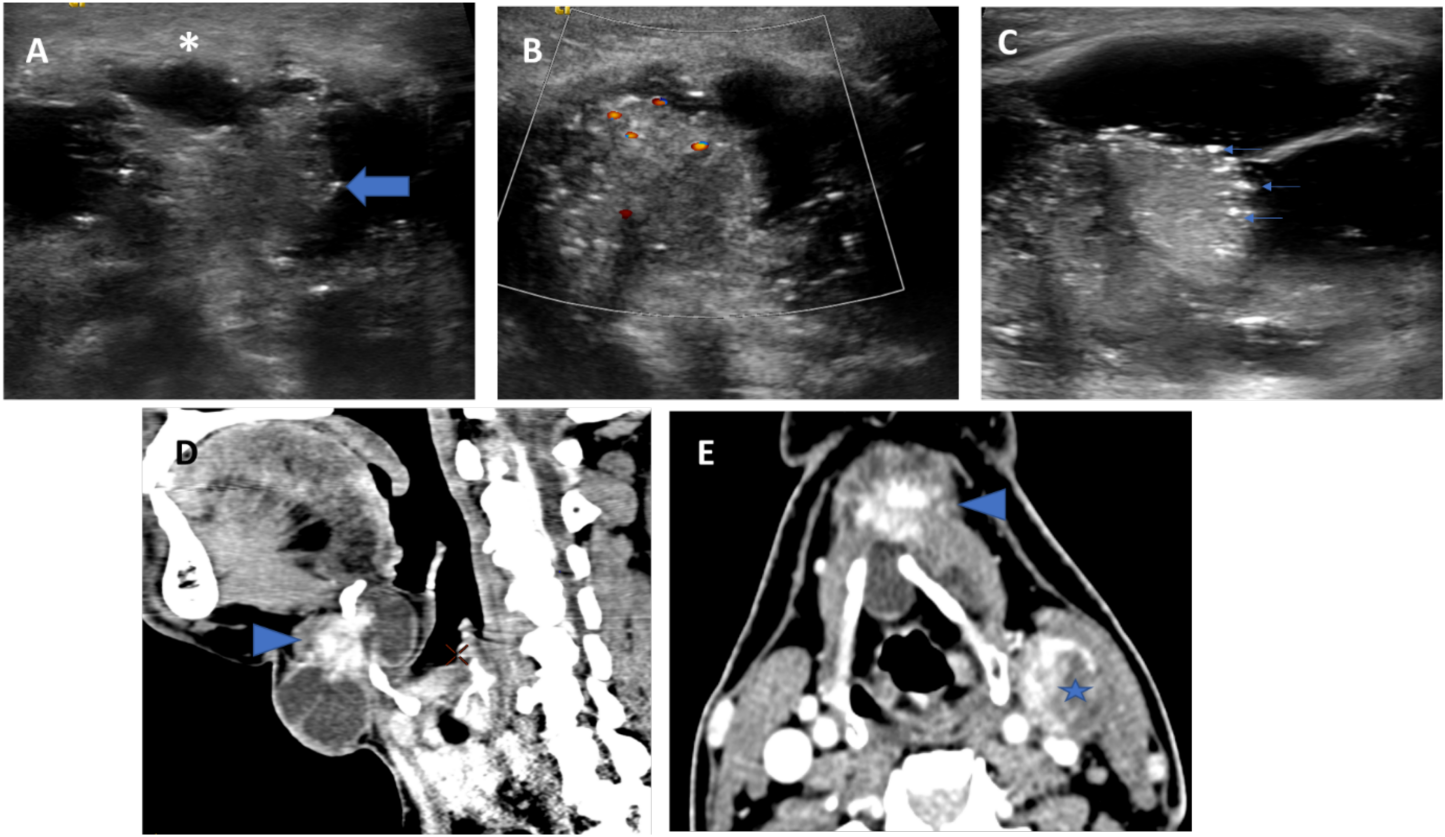
US **(A–C)** and CT **(D, E)** images of a histopathologically proven TGDC papillary carcinoma showing infrahyoid lesion with solid-cystic composition (thick arrow indicates the solid component) and irregular margins (asterisk). The solid component shows internal vascularity **(B)** and microcalcifications (thin arrows). CT showed irregular enhancing solid component and a metastatic left level III node (star).

US images were available for 21 patients with TGDC malignancy. On imaging studies, the malignant lesions ranged from 8 – 41 mm in the greatest dimension with an average size of 23.3 mm and a standard deviation of 11.5. The sonographic characteristics of malignant versus benign TGDC are shown in **Table 1**. Compared to their benign counterparts, malignant TGDCs were more likely to present with the following US characteristics: irregular or lobulated margins (90.4 vs. 38.10%), solid-cystic composition (61.90 vs. 17.07%), internal vascularity (47.62 vs. 4.88%), internal calcification (76.19 vs. 7.32%) (each p-value < 0.005) (**Figure 1**). However, there were no significant differences between benign and malignant TGDC in terms of the US features of size (p-value 0.08) and location (p-value = 0.55).

**Table 1:** US characteristics of malignant versus benign thyroglossal duct cysts.

Feature		Benign TGDC		Malignant TGDC		P-value
**Size**		Number	Percentage	Number	Percentage	
	< 15 mm	30	88.23%	4	11.76%	0.083
	> 15 mm	29	69.04%	13	30.95%	
**Location**						
	Suprahyoid	20	54.05%	17	45.94%	0.015
	Infrahyoid	21	84.00%	4	16.00%	
**Margins**						
	Irregular	8	29.62%	19	70.37%	0.0001
	Regular	33	94.28%	2	5.71%	
**Composition**						
	Solid-cystic	7	35.00%	13	65.00%	0.0001
	Cystic	34	80.95%	8	19.05%	
**Internal echoes**						
	Present	10	83.33%	2	16.66%	0.161
	Absent	31	62.00%	19	38.00%	
**Internal vascularity**						
	Present	2	16.66%	10	83.33%	0.0001
	Absent	39	78.00%	11	22.00%	
**Calcifications**						
	Present	3	15.78%	16	84.21%	0.0001
	Absent	38	88.37%	5	11.62%	

The sensitivity, specificity, PPV and NPV of ultrasound features for prediction of malignancy in TGDC are depicted in **Table 2**. The presence of calcifications and internal vascularity were the most specific imaging features for malignancy while the presence of irregular/lobulated margins had the highest sensitivity for the detection of malignancy. Purely cystic nodules with or without internal echoes favoured the benign nature of TGDC.

**Table 2:** Predictive value of US features of malignant thyroglossal duct cysts.

	Irregular margin	Solid-cystic composition	Internal vascularity	Calcification
**Sensitivity**	90.48%	61.90%	47.62%	76.19%
**Specificity**	61.90%	82.93%	95.12%	92.68%
**PPV**	70.37%	65.00%	83.33%	84.21%
**NPV**	86.67%	80.95%	78.00%	88.37%

A receiver operating characteristics (ROC) curve was calculated to validate the relationship between the US features and malignant TGDC. The area under the curve was 0.88, signifying that the accuracy of ultrasound as a diagnostic tool was good.

### CT Features

CT images were available for 6 patients with TGDC carcinoma. In most of these, the lesion appeared solid-cystic in composition, with enhancing solid component, irregular wall enhancement and nodularity (**Figure 1**). One case showed internal calcifications. The lesions showed no evidence of adjacent infiltration (**Table 3**).

**Table 3:** CT features of TGDC lesions.

CT Features		Diagnosis		Total	
		Benign	Malignant	Percentage	In valid P values
**Density**	**hypodense**	15	3	18	0.656
		83.3%	16.7%	100.0%	
	**hyperdense**	1	0	1	
		100.0%	0.0%	100.0%	
**Enhancement**	**Enhancement**	6	3	9	0.047
		66.7%	33.3%	100.0%	
	**No Enhancement**	10	0	10	
		100.0%	0.0%	100.0%	
**Calcifications**	**Present**	3	1	4	0.57
		75.0%	25.0%	100.0%	
	**Absent**	13	2	15	
		86.7%	13.3%	100.0%	
**Lymph node**	**Suspicious**	4	2	6	0.154
		66.7%	33.3%	100.0%	
	**Benign**	12	1	13	
		92.3%	7.7%	100.0%	
**Total**		16	6	22	
				100.0%	

### Thyroid gland imaging

**Figure 2:**
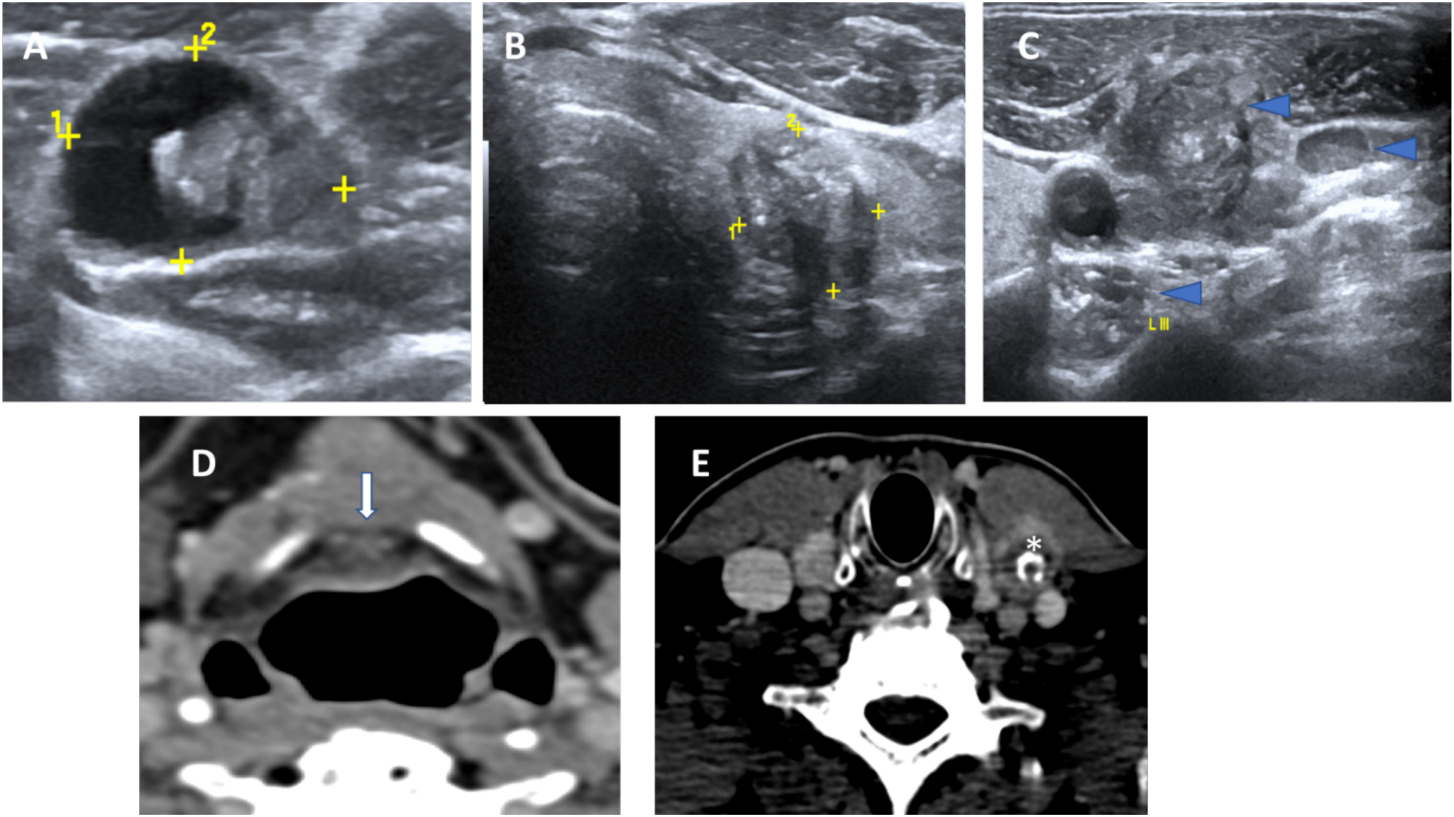
Concurrent histopathologically proven papillary carcinoma of the thyroid in a case of TGDC papillary carcinoma. US images showing a malignant TGDC containing solid component and microcalcifications **(A)** with synchronous left thyroid lobe papillary carcinoma **(B)** and multiple metastatic left sided nodes showing microcalcifications **(C)**. Corresponding CT images showing the midline TGDC papillary carcinoma (arrow in **D**) and metastatic left cervical node (asterisk in **E**).

A total of 12 out of the 22 patients (54%) had an associated papillary carcinoma of the thyroid gland; 11 of these were diagnosed on pre-operative US. These nodules appeared hypoechoic, solid-cystic/cystic with irregular margins, and absent/interrupted halo. Micro-calcifications were noted in 8 patients. They were assigned a category of ACR-TIRADS 5 (Thyroid Imaging Reporting and Data System by American College of Radiology) on ultrasound (**Figure 2**).

### Neck nodes

Out of 22 (50%) patients, 11 had positive neck nodes on histopathology of which 9 patients had a concurrent thyroid gland malignancy; while 2 had a papillary carcinoma confined to the TGDC. These were labelled suspicious on pre-operative imaging; 10 on US and 2 on CT examination. On US, suspicious lymph nodes appeared round, with loss of fatty hilum; and irregular margins with a heterogeneous echo texture, with or without micro-calcifications. On CT, the lymph nodes appeared round, revealed loss of reniform appearance, and showed calcifications and heterogeneous enhancement (**Figure 1**).

### Pathology findings

Pre-operative Fine needle aspiration cytology (FNAC) examination reports were available for 14 patients. Out of those 5 FNAC were diagnostic for papillary carcinoma, 3 samples were non-diagnostic and haemorrhagic aspirate, and 6 cases were proven as concurrent thyroid malignancy. In these cases, cytologic evaluation revealed clusters and papillae of thyroid follicular cells showing nuclear enlargement, grooving, overlapping and inclusions. A few clusters of metaplastic cells were also seen in a background of abundant thin colloid-like material. There were no false-negative cases. Histopathology confirmed the diagnosis of papillary carcinoma of TGDC in all cases.

### Treatment details

Out of 22 malignant TGDCa, 20 were operated (surgical details as summarized in the supplementary **Table 4**). Operative details were not available for 2 patients. Neck dissection was performed in 14 patients; out of the 14 neck dissections, neck nodes were positive in 11 cases. 7 patients received adjuvant treatment of radioactive iodine.

**Table 4:** Summary of the management details in our study.

Type of Surgery	Malignant	Benign
Excision	0	1
Sistrunk procedure	5	2
Total thyroidectomy with Sistrunk	7	0
Hemithyroidectomy with Sistrunk	2	0
Total thyroidectomy	6	2
Conservative management	0	59
Operative details not available	2	0
**Total**	22	64

### Follow up

All of the TGDC carcinoma patients were disease free till recent follow up or at the end of 5 years in applicable cases. In our study the mean time period of follow-up post-surgery of malignant TGDCa lesions was 49.88 months with inter quartal range of 7.84 - 65.33 months. Patients who underwent a total thyroidectomy with Sistrunk procedure were followed up 3-6 monthly in the first 2 years and then 6 monthly till 5 years from the completion of treatment and annually subsequently. They were followed up with ultrasound examination of the neck and thyroglobulin assay; while those who underwent an isolated Sistrunk procedure did not undergo thyroglobulin (Tg) testing. Patients who didn’t have clinical evidence of disease along with low Tg levels and no evidence of disease on US (if done) and a negative RAI scan (if done) were considered disease-free.

## Discussion

Thyroglossal duct cysts develop from persistent remnants of the thyroglossal duct at any site along the path of descent (1–3). The typical locations of TGDCs are between the thyroid gland and the hyoid bone (61%), i.e., infra-hyoid, followed by the suprahyoid (24%), suprasternal (13%) and rarely intralingual (2%) regions (1, 3). In our study, the supra-hyoid midline location was the commonest site, in TGDC carcinomas as well as in their benign counterparts. TGDC carcinomas arise from ectopic thyroid tissue (1–3). The vast majority of these belong to the papillary subtype accounting for as many as 85% of cases. Mixed papillary-follicular carcinomas and squamous cell carcinomas are uncommon and constitute the remainder (2, 3).

The average age of presentation of TGDC carcinoma has been reported as 38 years; the median age in our study was found to be 47 years ranging from 24 to 88 years. A higher prevalence has been observed in females, with a ratio of 3:2 in published literature (3); this was not seen in our study.

There is a paucity of radiology literature describing the imaging features for the definitive diagnosis of carcinoma in thyroglossal duct remnants. We encountered four imaging features on US which were found to be highly predictive of malignancy in TGDC; namely irregular/lobulated margins, solid-cystic composition, internal vascularity and presence of calcifications. The most commonly encountered feature was the presence of irregular/lobulated margins which was indicative of the invasive nature of the lesion, followed by solid-cystic composition. Our findings differed slightly from those seen in the case series by Choi et al. (3), which is one of the largest published series on imaging of TGDC malignancies. In their series of ten patients, eight cases presented with a solid-cystic nodule on imaging, which was the predominant imaging feature in their study.

The presence of calcifications within the solid component can be regarded as a highly specific feature of TGDC malignancy on US; this has not been established in prior studies. We did not encounter any case of histopathologically proven TGDC carcinoma without suspicious features on pre-operative imaging. Our findings differed from those in the study by Thompson et al., who encountered features suspicious for malignancy only in 9 out of 22 (41%) cases (4).

CT imaging of TGDC carcinomas revealed a heterogeneously enhancing cystic lesion located in the midline location with a solid component within. These findings concurred with those encountered by other investigators (5). Glastonbury et al. reviewed the imaging features of 6 cases of TGDC carcinoma on CT or MRI. They concluded that malignancy in a TGDC should be suspected in the presence of a solid component or invasive features within a TGDC (6). They also observed that the presence of calcification could be regarded as a specific marker for malignancy; and is best seen on CT imaging. CT imaging will be worthwhile in the following scenarios: **a.** In difficult cases to assess the entire tract using only US. **b.** For those patients who are unable to position themselves properly for US examinations**. c.** Compared to other common modalities, US evaluation requires specialized knowledge and operator skills. **d.** Accurate assessment of the depth of the lesion, especially the distance from the pharyngeal mucosa is important before TGDC surgery. **e.** It is also necessary to identify metastatic lesions in malignant cases. In our experience, US evaluation alone showed a good accuracy for the diagnosis of malignant TGDC. The US provided additional information with respect to the status of the thyroid gland and cervical lymphadenopathy.

The concomitant occurrence of papillary carcinoma in a TGDC and thyroid has been documented in the literature; with different occurrence rates across studies (3, 4, 7). According to Widström et al. the criteria for diagnosis of primary carcinoma of the thyroglossal duct includes the following: histological identification of TGDC by demonstration (i.e. epithelial lining of ducts with normal thyroid follicles within walls of the cysts), normal thyroid tissue adjacent to the tumour, and histopathological examination of the thyroid gland showing no sign of primary carcinoma (8). Since a co-existing thyroid carcinoma was seen in nearly 50% of cases in our study, we recommend that a careful sonographic evaluation of the thyroid gland for a suspicious nodule is of utmost importance to avoid missing out on a co-existent malignancy.

We observed that patients with a co-existing thyroid malignancy were more likely to have metastatic neck nodes, as compared to those with a TGDC malignancy alone. ‘‘Skip’’ metastases to the lateral compartment (levels III and IV), with no central compartment (level VI) metastases, were found in as many as 45.4% cases with nodal involvement. This was also seen in the study by Hartl et al. (9) who encountered skip metastatic nodes in 40% patients. This could occur as the lymphatic drainage of TGDCs occurs preferentially to the lateral neck nodes rather than to the central compartment nodes (10). The occurrence of skip neck nodes may not however be a prognostic factor in TGDC carcinomas; it is believed that the number of metastatic nodes and the presence of peri-nodal extension could have an impact on prognosis; as demonstrated in thyroid carcinomas (10). In our study, a 5 year follow-up was available in all patients with metastatic neck nodes; however, no evidence of disease recurrence was seen in any of these patients after surgical management. A larger series of patients with a longer duration of follow up are necessary to evaluate their impact on disease prognosis.

According to published literature, FNAC has a relatively high false-negative rate of 47% in diagnosing papillary carcinoma in TGDC with a modest true positive rate of 53% (11). Inadequate cellularity secondary to dilution by the cystic contents could be responsible for the high false-negativity of FNAC; thus, it is advisable to target the solid component under US guidance after aspiration of the cystic contents (11). In our experience, imaging plays a strong complementary role to FNAC. The accuracy rate of benign or malignancy by FNAC is not high enough, but the positive predictive value is very high, making it a strong basis for deciding treatment strategies in positive cases. FNAC may aid the diagnosis but a negative result cannot exclude the possibility of a malignancy as inconsistent results are frequent due to sampling errors.

## TGC-TIRADS classification for thyroglossal cyst lesions

Based on the results of our study, we now propose a modified version of the “TGC-TIRADS” classification for the preoperative diagnosis and risk stratification of thyroglossal cyst lesions. This classification is based on the analysis of USG features, including the composition of the lesion, margins, presence of internal vascularity, internal echoes, and calcifications. The proposed TGC-TIRADS classification is as follows:

### TGC-TIRADS 1

Absence of remnant tissue along the thyroglossal tract.

### TGC-TIRADS 2

Purely cystic thyroglossal lesion.

### TGC-TIRADS 3

Thyroglossal cyst with internal echoes.

### TGC-TIRADS 4A

Thyroglossal cyst with any one of the features:

- Solid cystic in composition.
- Presence of calcifications.
- Presence of internal vascularity.
- Presence of irregular margins.

### TGC-TIRADS 4B

Thyroglossal cyst with any two of the above-mentioned features.

### TGC-TIRADS 4C

Thyroglossal cyst with any three of the above-mentioned features.

### TGC-TIRADS 5

Thyroglossal cyst with all of the four above-mentioned features.

**Figure 3:**
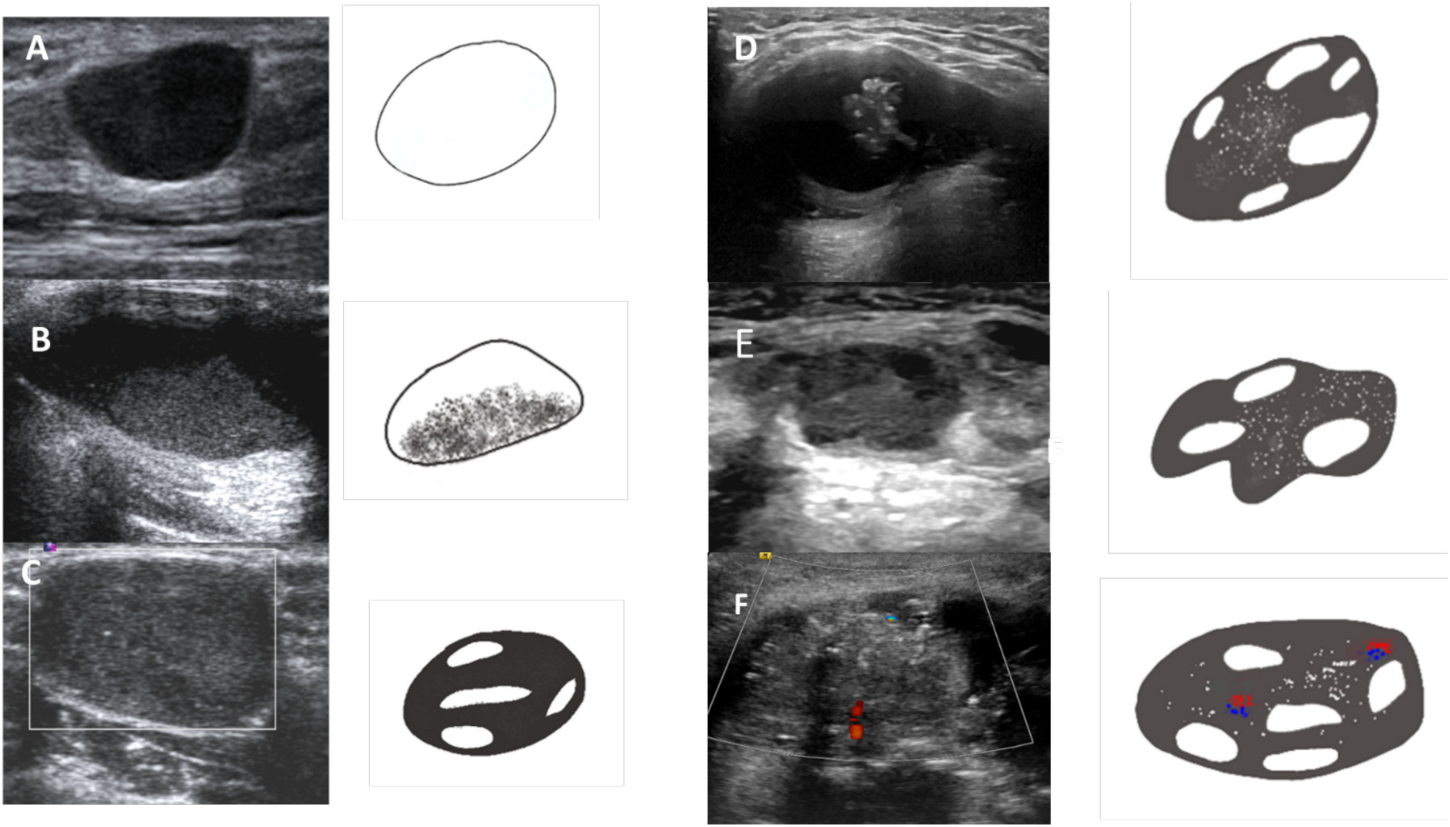
Lesions corresponding to various TGC-TIRADS categories based on the US features. **(A)** TGCTIRADS 2: Purely cystic thyroglossal lesion. **(B)** TGC-TIRADS 3: Thyroglossal cyst with internal echoes. **(C)** TGC-TIRADS 4A: Well defined solid cystic lesion without internal vascularity or calcification. **(D)** TGC-TIRADS 4B: Well defined solid cystic lesion with internal calcifications. **(E)** TGC-TIRADS 4C: Solid cystic lesion with irregular margins and internal calcifications. **(F)** TGC-TIRADS 5: Solid cystic lesion with irregular margins, internal calcifications and internal vascularity.

**Figure 3** shows US features of various of the proposed TGC-TIRDADS categories.

**Table:** 

Category	Implication	Recommendation
**TGC-TIRADS 1**	Normal	No follow-up needed
**TGC-TIRADS 2**	Benign	No FNA required
**TGC-TIRADS 3**	Probably benign	No FNA required
**TGC-TIRADS 4A**	Undetermined	FNA recommended*
**TGC-TIRADS 4B**	Moderate suspicious	FNA recommended*
**TGC-TIRADS 4C**	Highly suspicious	FNA recommended*
**TGC-TIRADS 5**	Consistent with malignancy	FNA recommended*

*As sampling errors frequently produce conflicting results, imaging features plays a strong complementary role to cytologic examination. Although FNAC may help with the diagnosis, a negative test does not rule out the possibility of cancer.

**Table 5** provides statistics for the assigned TGC-TIRADS category and the percentage likelihood of malignancy for each category as seen in our study.

**Table 5:** Statistics of the assigned TGC-TIRADS category and their respective percentage likelihood of malignancy as observed in our study.

Category	Total cases		Benign		Malignant	
	Number	Percentage	Number	Percentage	Number	Percentage
**TGC-TIRADS 2**	17	27%	17	100%	0	0%
**TGC-TIRADS 3**	9	15%	9	100%	0	0%
**TGC-TIRADS 4A**	15	24%	11	73%	4	27%
**TGC-TIRADS 4B**	6	10%	3	50%	3	50%
**TGC-TIRADS 4C**	8	13%	1	12%	7	88%
**TGC-TIRADS 5**	7	11%	0	0%	7	100%

### Management

Surgical management of a benign TGDC involves the Sistrunk procedure, based on the embryonic development of the lesion. This includes the excision of the cyst and the tract of descent, extending from the foramen cecum at the base of the tongue, along with the mid-segment of the hyoid bone (1). It has been observed that the only significant prognostic predictor of overall survival in patients with TGDC carcinoma is the extent of surgery for the TGDC (12). There is a widespread consensus that the Sistrunk procedure is the minimum that is required for the management of localized TGDC carcinomas (13). The need to resect a clinically and radiologically normal thyroid gland in the setting of a TGDC carcinoma is controversial.

Plaza et al. proposed that an isolated Sistrunk surgery would suffice for papillary carcinoma in TGDC, for patients below 45 years of age with tumour size below 15 mm and a normal thyroid gland on US with no suspicious neck nodes. They recommended a total thyroidectomy with neck dissection only if lymph node metastases were found on ultrasound or during surgery; followed by radioiodine therapy (14). Some investigators have recommended that a total thyroidectomy must be performed in all patients with TGDC carcinoma, as it could decrease the risk of disease recurrence in future (15, 16). In our experience, the benefit of performing a total thyroidectomy in addition to the Sistrunk procedure, in the absence of a suspicious thyroid lesion is questionable. Any suspicious nodule in the thyroid gland suggestive of multicentric disease, the presence of suspicious or cytologically metastatic nodes, aggressive variants, or disease involving the thyroid lobe led to the consideration of total thyroidectomy. Such a synchronous thyroid lesion may then represent either two independent primary carcinomas, a metastasis from a primary TGDC carcinoma or a primary thyroid carcinoma with metastasis to the thyroglossal duct (14). Similar recommendations were made by Thompson et al. (4), Doshi et al. (17) and Zhu et al. (18) in their respective studies.

The occurrence of metastatic cervical nodes in cases of papillary carcinoma of the TGDC is reported to be 7 to 15% (7, 12). Dzodic et al. (19) found that the extent of lymph node dissection has an impact on the prognosis of TGDC cancer. We observed that there is no role for elective neck dissection in patients with an isolated TGDC malignancy without suspicious neck nodes. This recommendation concurs with that of Patel et al. who analyzed the prognostic factors for TDGC carcinoma and concluded that neck node dissection did not have a significant impact on prognosis (12). Selective nodal dissection with an intra-operative frozen section may be performed for sonologically suspicious nodes in isolated TGDC carcinomas. Central compartment clearance with modified radical neck dissection is indicated only in patients with a co-existing thyroid malignancy with suspicious neck nodes on imaging.

**Figure 4:**
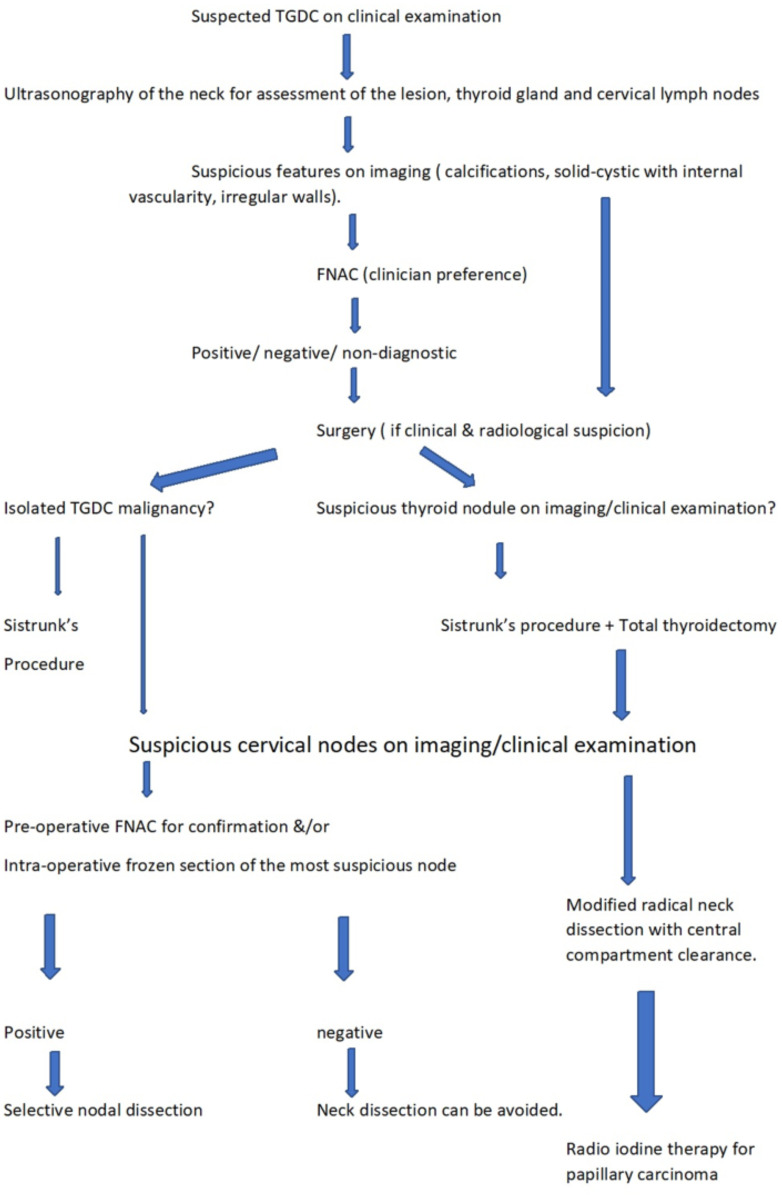
Flowchart showing recommended approach for the diagnosis and management of thyroglossal duct cyst carcinomas based on our study.

Luna-Ortiz et al. suggested a risk stratification approach and recommended that I^131^ therapy was indicated in older patients, those with metastatic disease, high-risk histological features, patients with a prior head and neck irradiation or those with a co-existing thyroid gland malignancy (20). This has also been largely accepted by other authors (21). Based on our experience, we recommend that only the presence of a co-existing thyroid gland papillary carcinoma necessitates the administration of radioiodine therapy. All cancers were considered for RAI except tumours <1 cm with no high-risk features as per ATA guidelines 2009. The dose varied with the risk category. However, with the revised guidelines ATA 2015 only intermediate and high risk of recurrence cases were considered for RAI. The development of risk stratification systems would require further studies with large sample sizes to determine the statistical significance of various prognostic indices. **Figure 4** provides an overview of the recommended approach for the diagnosis and management of thyroglossal duct cyst carcinomas based on our study.

### Follow-up and prognosis

All 22 patients were followed up at our institute, and all were found disease-free at the end of 5 years. Similarly, in the study by Heshmatl et al., 12 operated cases of TGDC carcinomas were found to be disease-free during a mean follow-up interval of 13 years (7). The prognosis associated with TGDC papillary carcinoma is reported to be excellent, with a very low incidence of recurrence (22); also seen in our study.

Our study had two major limitations. In differentiated carcinomas derived from thyroid tissue, cases with very slow progression were included. Pathological diagnosis by surgical resection was not performed in all cases. Therefore, diagnosis through cytology or follow-up alone may include malignancy within the non-malignant group. Also, the significance of treatment methods and prognosis of this study is particularly limited.

## Conclusion

USG of the neck is the imaging of choice for the diagnosis of TGDCa. The presence of irregular/lobulated margins, solid-cystic composition, internal vascularity, and calcifications are reliable US predictors of malignancy. The pre-operative diagnosis and risk stratification of thyroglossal lesions will be aided by the application of the proposed TGC-TIRADS classification, for which the percentage likelihood of malignancy correlated well with the results in our study. Additionally, it is important to evaluate the thyroid gland for a synchronous malignancy and the neck for metastatic lymphadenopathy, in order to formulate a plan of management. The sistrunk procedure is adequate for patients with non-metastatic TGDC in the absence of an associated thyroid malignancy. Patients with suspicious neck nodes on imaging could require selective nodal dissection in addition to the Sistrunk procedure. Total thyroidectomy with neck dissections is necessary in the presence of a co-existing thyroid malignancy. With appropriate surgery, TGDC papillary carcinomas tend to have an excellent prognosis with a low incidence of recurrence.

## Data availability statement

The raw data supporting the conclusions of this article will be made available by the authors, without undue reservation.

## Author contributions

AM and SD conceived and designed the structure of this manuscript. AM, SD and SSu wrote the paper. AM, SD, SSu, SSh and UA revised the paper. All authors contributed to the article and approved the submitted version.
